# Detection of extended spectrum beta-lactamase genes in *Pseudomonas aeruginosa* isolated from patients in rural Eastern Cape Province, South Africa

**DOI:** 10.1038/s41598-021-86570-y

**Published:** 2021-03-29

**Authors:** Mojisola C. Hosu, Sandeep D. Vasaikar, Grace E. Okuthe, Teke Apalata

**Affiliations:** 1 Division of Medical Microbiology, Department of Laboratory Medicine and Pathology, Faculty of Health Sciences, Walter Sisulu University and National Health Laboratory Services, Mthatha, Eastern Cape South Africa; 2 Department of Biological and Environmental Sciences, Walter Sisulu University, Mthatha, Eastern Cape South Africa

**Keywords:** Microbiology, Medical research, Molecular medicine

## Abstract

The proliferation of extended spectrum beta-lactamase (ESBL) producing *Pseudomonas aeruginosa* represent a major public health threat. In this study, we evaluated the antimicrobial resistance patterns of *P. aeruginosa* strains and characterized the ESBLs and Metallo- β-lactamases (MBL) produced. Strains of *P. aeruginosa* cultured from patients who attended Nelson Mandela Academic Hospital and other clinics in the four district municipalities of the Eastern Cape between August 2017 and May 2019 were identified; antimicrobial susceptibility testing was carried out against thirteen clinically relevant antibiotics using the BioMérieux VITEK 2 and confirmed by Beckman autoSCAN-4 System. Real-time PCR was done using Roche Light Cycler 2.0 to detect the presence of ESBLs; *bla*_SHV_, *bla*_TEM_ and *bla*_CTX-M_ genes; and MBLs; *bla*_IMP_, *bla*_VIM._ Strains of *P. aeruginosa* demonstrated resistance to wide-ranging clinically relevant antibiotics including piperacillin (64.2%), followed by aztreonam (57.8%), cefepime (51.5%), ceftazidime (51.0%), piperacillin/tazobactam (50.5%), and imipenem (46.6%). A total of 75 (36.8%) multidrug-resistant (MDR) strains were observed of the total pool of isolates. The *bla*_TEM_, *bla*_SHV_ and *bla*_CTX-M_ was detected in 79.3%, 69.5% and 31.7% isolates (n = 82), respectively. The *bla*_IMP_ was detected in 1.25% while no *bla*_VIM_ was detected in any of the strains tested. The study showed a high rate of MDR *P. aeruginosa* in our setting. The vast majority of these resistant strains carried *bla*_TEM_ and bla_SHV_ genes. Continuous monitoring of antimicrobial resistance and strict compliance towards infection prevention and control practices are the best defence against spread of MDR *P. aeruginosa*.

## Introduction

*Pseudomonas aeruginosa* is an opportunistic pathogen causing infections especially in immunocompromised patients. It is the leading cause of nosocomial infections such as urinary tract infections, surgical site infections, pneumonia, bacteremia and septicaemia^1,2^. It is one of the ESKAPE pathogens that is most medically and epidemiologically significant and has been implicated as a principal cause of chronic lung infections in cystic fibrosis (CF) patients and severe infections in burn victims^3,4^. The World Health Organization (WHO) has categorized *P. aeruginosa* as a critical priority pathogen, which needs urgent novel antibiotics intervention and was given a serious threat level due to multidrug resistance displayed to many antibiotics^5,6^. The growing resistance of *P. aeruginosa* to several antibiotics, as a result of excessive antibiotic administration, has resulted to the accumulation of antibiotic resistance and cross-resistance between antibiotics and the advent of multidrug-resistant (MDR) forms of *P. aeruginosa*. *P. aeruginosa* infections are generally linked with high mortality; this is due to its innate resistance to several antimicrobial agents and acquired resistance via mutation and horizontal transfer^7,8^ Various mechanisms involved in the resistance of *P. aeruginosa* include over expression of efflux pump, acquisition of Extended-Spectrum β-Lactamases (ESBLs) and Metallo-β-Lactamases (MBLs)^9^. ESBLs are a cluster of β-lactamases that inactivates β-lactams especially oxymino-β-lactams and monobactams, and are repressed by β-lactamase inhibitors, such as clavulanic acid. They are encoded on plasmids and can easily be conveyed from one organism to another^10^. ESBL enzymes according to Ambler classification are categorized into two, A and D. The most prevalent enzymes in class A include *bla*_TEM_, *bla*_CTX-M_ and *bla*_SHV_, and has been described in *P. aeruginosa* strains^10,11^. The emergence of beta-lactamase enzymes is majorly due to chromosomal mutation and procurement of resistance genes which are moved about on various mobile genetic elements (MGEs) such as—bacteriophages, genomic islands, integrons, plasmids and insertion sequences^12^. The production of these enzymes is a going concern for infection control supervision because it restricts therapeutic choices. Continuous monitoring and timely detection of ESBL and MBL producing organisms is critical to establish suitable antimicrobial therapy and to thwart their spread^13^. Polymerase chain reaction (PCR)-based methods are critical to establish the prevalence and characterization of beta lactamases due to the presence of multiple resistance genes in some microorganisms^14^. Real-time PCR (rPCR) detection of ESBL enhances faster diagnosis and timely management of epidemiological information for monitoring outbreak situations^15^. Studies on ESBL-producing *P. aeruginosa* in South Africa have been documented from other provinces^16–18^ but scarce data exist in the Eastern Cape particularly in the former Transkei region on the molecular detection of ESBLs and MBLs in *P. aeruginosa*.

Antibiotic surveillance studies are important for the design of control strategies for preventing bacterial resistance and establishing therapeutic guidelines as well as for a better understanding of bacterial epidemiology. The first reported National antimicrobial resistance (AMR) surveillance in South Africa^19^ reported ESKAPE organisms causing bacteremia hence not much data for -comparative analysis. The data from the comprehensive view of AMR in blood cultures for ESKAPE pathogens revealed that 20% and 25% of *P. aeruginosa* bloodstream isolates were resistant to piperacillin/tazobactam and carbapenems respectively. To the best of our knowledge, there are few reports on surveillance of antimicrobial resistance (AMR) in clinical isolates of *P. aeruginosa* from all samples obtainable particularly from the Eastern Cape. The aim of this study was to examine the antimicrobial susceptibility profiles of clinical strains of *P. aeruginosa* obtained from patients attending healthcare facilities in the four district municipalities in Eastern Cape and to investigate their ESBL and MBL resistance mechanisms.

## Results

### Identification of P. aeruginosa and demographics

During the study period, a total of 204 *P. aeruginosa* isolates were identified from a range of clinical specimens of patients who attended various healthcare facilities in the OR Tambo district municipality, Alfred Nzo, Joe Gqabi and Amathole districts in the Eastern Cape Province. The strains were identified by Vitek 2 system (bioMérieux, Inc., USA), and confirmed by both Microscan autoscan-4 system (Beckman Coulter, Inc. USA) and rPCR using specific primer and probes targeting *gyr*B. The majority of the strains were from male patients (60%) while 40% belonged to female patients. The strains were predominantly from pus and wound swabs (80.4%), with surgical wounds constituting 43.3%, burn wounds 3.7% and others accounted for 53.0%. These samples originated from Surgical (33.3%), General (18.1%) and Paediatrics (11.3%) wards.

The mean age of patients was 32.8 years ranging from 6 days to 84 years. The male population have a mean age of 30.5 years ranging from 6 days to 83 years while the female mean age was 36.2 years with age ranging from 22 days to 84 years. These patients were drawn from four district municipalities with OR Tambo having the most at 80.4% with the least patient drawn from Joe Gqabi at 0.5% (Table 1).

**Table 1:** General characteristics of study population.

Variable	Number, n (%)
**Gender**	
Male	122 (59.8)
Female	82 (40.2)
**Age (years) (mean ± SEM)**	
≤ 15 (7.4 ± 0.6)	44 (21.6)
16–30 (23.9 ± 0.7)	48 (23.5)
31–45 (37.2 ± 0.6)	44 (21.6)
46–60 (54.1 ± 0.8)	30 (14.7)
> 60 (71.7 ± 1.3)	21 (10.3)
**Specimen source**	
Pus and wound swab	164 (80.4)
Sputum	22 (10.8)
Fluid aspirate	7 (3.4)
Catheter tip	6 (2.9)
Urine	3 (1.5)
Tissue	2 (1.0)
**MDR status**	
MDR	75 (36.8)
Non-MDR	129 (63.2)
**District municipality**	
Alfred Nzo	27 (13.2)
dAmathole	12 (5.9)
Joe Gqabi	1 (0.5)
OR Tambo	164 (80.4)

### Antimicrobial susceptibility

**Figure 1:**
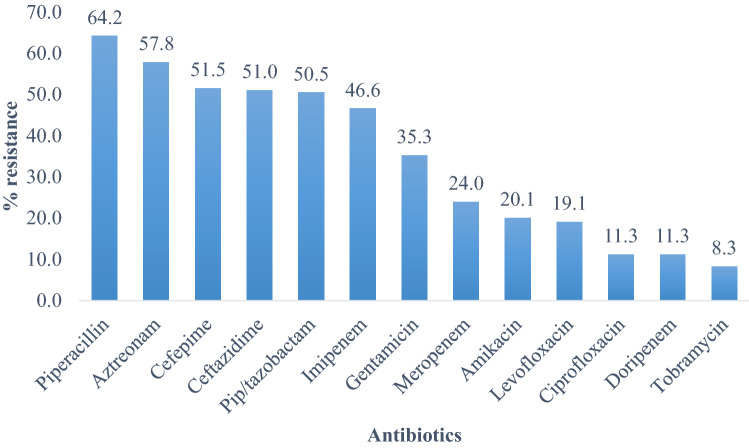
Antibiotic resistance pattern of the *P. aeruginosa* isolates (N = 204).

Out of 204 isolates tested to various antibiotics, there was resistance observed in piperacillin (64.2%), followed by aztreonam (57.8%), cefepime (51.5%), ceftazidime (51.0%), piperacillin/tazobactam (50.5%), and imipenem (46.6%). Other percentages of resistance included gentamicin (35.3%), meropenem (24.0%) and amikacin (20.1%). Tobramycin was the most potent antibiotic with susceptibility of 91.7% followed by both doripenem and ciprofloxacin (88.7%) and levofloxacin (80.1%) (Fig. 1). The study also revealed a total of seventy-five isolates (36.8%) were multidrug-resistant out of the tested strains of which the majority was drawn from the OR Tambo district municipality (82.7%), Alfred Nzo (12%) and the least from Amathole (5.3%); while non-MDR constituted 63.2% of the total.

### Molecular Detection of ESBL- and MBL-encoding genes

Of 204 *P. aeruginosa* isolates, 82 were tested by singleplex rPCR for detection of ESBL and MBL. These data revealed that ESBL—genotypic resistance is driven by *bla*_TEM_ (79.3%) followed by *bla*_SHV_ (69.5%) and lastly *bla*_CTX-M_ (31.7%). MBL-genotypic resistance, *bla*_VIM_, was not detected in all strains tested while only one *bla*_IMP_ (1.25%) was detected (Table 2). The most common ESBL-genotype combination among the *P. aeruginosa* was a combination of *bla*_TEM_ + *bla*_SHV_ (40.5%).

**Table 2:** ESBL genotypes in *P. aeruginosa* strains.

Positive by PCR for ESBL genes	Number amplified (N = 82)	Total (%)
**A. Single ESBL gene**		
*bla*_TEM_	65	79.3
*bla*_SHV_	57	69.5
*bla*_CTX-M_	26	31.7
*bla*_IMP_	1	1.25
*bla*_VIM_	0	0
**B. Two or more ESBL genes**	121	
*bla*_TEM_ + *bla*_SHV_	49	40.5
*bla*_TEM_ + *bla*_CTX-M_	22	18.2
*bla*_SHV_ + *bla*_CTX-M_	26	21.5
*bla*_TEM_ + *bla*_IMP_	1	0.8
*bla*_TEM_ + *bla*_SHV_ + *bla*_CTX-M_	22	18.2
*bla*_TEM_ + *bla*_SHV_ + *bla*_IMP_	1	0.8
*bla*_TEM_ + *bla*_SHV_ + *bla*_CTX-M_ + *bla*_IMP_	0	0

## Discussion

The current study revealed that antibiotic resistance was observed in piperacillin (64.2%), aztreonam (57.8%), Cephalosporins (cefepime 51.5% and ceftazidime 51.0%), antipseudomonal penicillins + β-lactamase inhibitor (piperacillin/tazobactam 50.5%) and imipenem (46.6%). (Fig. 1). Others included gentamicin (35.3%), meropenem (24.0%) and amikacin (20.1%). Tobramycin was found to be the most potent antibiotic with a susceptibility of 91.7% followed by both doripenem and ciprofloxacin (88.7%) and levofloxacin (80.9%). Data from surveillance on isolates of *P. aeruginosa* in the South African public sector is not in agreement with the present study^20^. They reported much lower resistance rates of 15%, 16%, 24%, 23% and 19% in cefepime, ceftazidime, imipenem, meropenem and piperacillin/tazobactam respectively. The isolates were recovered from blood cultures only, possibly this might account for the variations in resistance rate, alternatively this might be due to regional variations in the empirical use of these antimicrobials^20^. This also underscores the importance of continuous local antimicrobial resistance surveillance for appropriate antibiotic treatment recommendations at the local and facility level since the data only accounted for bacteremic isolates, as there is no national surveillance data to facilitate comparison. In this study, the percentage of resistance of 11.3% to ciprofloxacin was within the same range of 13.4% described by Ramsamy et al.^18^. The data obtained were from nine public sector hospitals in KwaZulu-Natal Province. Additionally, gentamicin resistance of 17% and imipenem resistance of 13% as reported in the study was lower in comparison with resistance reported in the current study at 35.3% and 46.6% respectively. The susceptibility ranges of 75%-92% of *P. aeruginosa* isolates in this study to some routine antibiotics considered for therapy is encouraging but the increase in resistance exhibited to cephalosporins and imipenem is concerning. This might be due to selective pressure to those antibiotics and it will be important to monitor the prescription of these antibiotics. Owing to endless alteration, resistance exhibited to range of β-lactam antibiotics is challenging, thus making β-lactamase production the commonest cause of drug resistance and antimicrobial treatment failure^7,21^. This study detected an average resistance of 51.3% to the cephalosporins (ceftazidime and cefepime). Piperacillin and gentamicin resistance was 64.2% and 35.3% respectively similar to the findings of Uc-Cachon et al.^22^. The emerging level of resistance displayed to the cephalosporins highlight the development of cephalosporinases among resistant strains of these organisms. The cephalosporins due to their wide spectrum of activity are a significant class of antimicrobials used in controlling several infections however; the emergence of cephalosporinases can in effect hamper their clinical usefulness^23^. The reported increasing penicillinase-producing β-lactamases strains among these organisms validates the noticeably observable high rate resistance of our isolates to piperacillin^23^. Piperacillin is a penicillin beta-lactam antibiotic with in-vitro activity against Gram-positive and Gram-negative aerobic and anaerobic bacteria but because it is prone to hydrolysis by β-lactamase enzymes, its combination with tazobactam, a β-lactamase inhibitor, enhances the in-vitro activity of piperacillin to bacterial cells. This was noticed in the differences in the resistance to the two antibiotics with piperacillin having a higher resistance of 64.2% as compared to 50.5% in piperacillin/tazobactam.

Antibiotic resistance is a public health menace with an alarming proportion that is receiving collective attention more so that several studies have found a correlation between level of antibiotic prescription with the prevalence of antibiotic resistance^24,25^. Patients with resistant *P. aeruginosa* infections have a poor prognosis hence it is imperative that *P. aeruginosa* strains presenting severe drug resistance is monitored^26^. The swift spread and the emergence of MBL- and ESBL-producing *P. aeruginosa* of clinical origin is distressing and of great threat. Furthermore, level of antibiotic usage, horizontal gene transfer (HGT) event and environmental factors may account for variations in resistance patterns among strains isolated from diverse countries and regions. In the present study, 36.8% of the strains were MDR (defined as non-susceptibility to at least one agent in three or more antimicrobial categories). Studies have indicated that multidrug resistance often results into limited treatment options and adverse clinical and economic outcomes^27,28^.

Antimicrobial treatment is further hampered by the production of extended spectrum beta-lactamases and metallo beta-lactamases. The emergence of ESBL-producing *P. aeruginosa* is increasingly reported as a major cause of health-care associated infections. In the hospital locale, infections resulting from these resistant organisms are increasingly challenging to treat due to the intensity of resistance exhibited to the most commonly recommended antibiotics^14,29^. This study found out that the most prevalent genotype for ESBL production was *bla*_TEM,_ which was detected in 65 (79.3%) strains followed by *bla*_SHV_ (69.5%) and *bla*_CTX-M_ (31.7%) (Table 2). It has been reported that ESBL genes show variation depending on the geographical location, the findings of Erhlers et al.^30^, Chen et al.^31^ and Miranda et al.^32^, from South Africa, China and Brazil respectively corroborated our results of the prevalent genotype as *bla*_TEM_ while in contrast Jamali et al.^33^ reported the prevalent gene to be *bla*_SHV_. The predominant ESBL and MBL genes detected in a study conducted in Durban on MDR *P. aeruginosa* isolates were GES-2, OXA-21, and VIM-2^34^. The least detected ESBL genotype from this study was *bla*_CTX-M_ (31.7%) similar to Miranda et al.^32^. Although no *bla*_VIM_ was detected in our study, MDR *P. aeruginosa* encoding *bla*_VIM-2_ gene have been reported in a tertiary hospital in Cape Town, which was responsible for an outbreak, and in a public hospital in Port Elizabeth^35,36^. The phenotypic resistance displayed to the carbapenems particularly imipenem which is not validated by the genotypic MBL result may be due to other resistance mechanism such as efflux over expression or forfeiture of exterior membrane protein^37^.

Several researchers have reported on the concurrence of different β-lactamase genes found in the same strains^38,39^. The most common ESBL combination in this study was a combination of *bla*_TEM_ + *bla*_SHV_ (40.5%) contrary to Chen et al.^31^, who reported the commonest to be *bla*_SHV_ + *bla*_CTX-M_. The second most common genotype combination was *bla*_SHV_ + *bla*_CTX-M._ This study showed the most predominant ESBL gene was *bla*_TEM_, which is corroborated by other studies. Prior to now, *bla*_TEM_ used to be the most prevalent but recent reports suggest that the CTX-M-type group of ESBLs may now be the most predominant type globally^40^. These discrepancies may be due in part to varied geographic location, different levels of healthcare facilities involved, varied levels of exposure to healthcare settings, antibiotic use and antibiotic stewardship practices.

## Conclusions

This study is the first surveillance report on antimicrobial susceptibility testing and molecular detection of resistant genes of *P. aeruginosa* strains from clinical samples of patients attending healthcare facilities in four district municipalities of Eastern Cape Province, South Africa. The study showed a high rate of MDR *P. aeruginosa* in our setting. The vast majority of these resistant strains carried *bla*_TEM_ and bla_SHV_ genes. Early detection and characterization of ESBLs is critical to contain their dissemination, prevent outbreak and optimise therapy. Continuous monitoring of antimicrobial resistance and strict compliance towards infection control practices are the best defence against continuous spread of MDR *P. aeruginosa*.

The limitation of this study is the fact that we could not screen *P. aeruginosa* isolates for the presence of all reported genes (GES-2 and OXA-21 genes) from other South African provinces due to funding constraints.

## Methods

### Study design and settings

A prospective, cross-sectional descriptive study. Samples from patients were collected from August 2016 to May 2019. All methods were performed in accordance with the relevant guidelines and regulations and ethics approval certificate from the Faculty of Health Sciences Human Research Committee at Walter Sisulu University was obtained, bearing the registration number 024/2016. This laboratory-based study involved collection of a range of clinical specimens of patients who attended various healthcare facilities in the OR Tambo district municipality, Alfred Nzo, Joe Jqabi and Amathole districts in the Eastern Cape Province. Five local municipalities (King Sabata Dalindyebo, Nyandeni, Mhlontlo, Port St Johns, and Ingquza Hill) form the OR Tambo district municipality with an estimated total population of 1,760,389. Patients’ clinical samples were collected from 1 Academic Central Hospital (Nelson Mandela Academic Hospital), 1 Regional Hospital (Mthatha regional hospital), 12 District Hospitals; and 11 Community Health Centers. These samples were sent for culture and susceptibility testing in the Department of Medical Microbiology at the National Health Laboratory Services (NHLS), located in the Nelson Mandela Central Hospital in Mthatha, Eastern Cape. Clinical samples from those various hospitals and clinics were sent as part of the patients’ routine standard of care.

### Specimen collection and analysis

Non-duplicate *P. aeruginosa* isolates were collected from clinics and hospitals from the four district municipalities. Specimens included throat swabs, wound swabs, swabs from abscesses, sputum, urine, blood culture and catheter tips. Demographic characteristics of patients and medical histories were collected from medical records including date of specimen collection, gender and age. All samples were routinely cultured on MacConkey and Blood agar plates. Blood and sputum were also cultured on chocolate agar. Suspected colonies were plated on Cetrimide agar and identified by gram staining, colony characteristics, motility, pyocyanin production and characteristics grape-like odour^41^. Strains were identified to the species level with Vitek 2 GN (bioMérieux, Inc. USA) ID cards and confirmed by Microscan NID 2 panels (Beckman Coulter, Inc. USA). Specific primers and probes targeting *gyrB* were amplified by singleplex *rPCR* and were also used to confirm identity of the isolates.

### Antimicrobial susceptibility

Antimicrobial susceptibility was obtained by determining MIC using Microscan dehydrated broth microdilution method with negative MIC Panel Type 44 (NM44) (Beckman Coulter, Inc. USA) following the manufacturer’s guidelines^42^ MICs were interpreted following CLSI guidelines (M100-S27 breakpoints)^43^. The following antibiotics were tested in the panels: amikacin, aztreonam, cefepime, ceftazidime, ciprofloxacin, doripenem, gentamicin, imipenem, levofloxacin, meropenem, piperacillin/tazobactam, piperacillin and tobramycin.

### Criterion for multidrug resistance

The classification of MDR was performed according to Magiorakos et al*.*^44^. (MDR was defined as non-susceptibility to at least one agent in three or more antimicrobial categories).

### Molecular ESBL and MBL detection by singleplex rPCR

Genomic DNA was extracted using Roche MagNA Pure Bacteria lysis buffer, MagNA Pure Compact Nucleic Acid Isolation kit and PCR grade water (Roche Applied Science, Indianapolis), following manufacturer’s instructions. The DNA was used as a template in the rPCR analysis. Real time PCR for *bla*_CTX-M_, *bla*_SHV_, *bla*_TEM_, *bla*_IMP_ and *bl*a_VIM_ was carried out in the Light Cycler 2.0 instrument (Roche Applied Science, Germany) using Fast start Light Cycler 480 Hybridization probes Master kit (Roche Diagnostics, USA). The choice of testing for these genes was the result of scarcity of data in our setting. Specific primers and probes (Table 3) targeting the genes *bla*_CTX-M_, *bla*_SHV_, *bla*_TEM_, *bla*_IMP_ and *bl*a_VIM_ were amplified by singleplex rPCR. Primers were designed by TIB-Molbiol (Berlin, Germany). rPCR assay was performed in a 32 carousels using 20 µL capillaries with each capillary containing a total volume of 20 µL including 2 µL of Light Cycler FastStart DNA Master Hybridization Probe, 2 µL of primers (0.5 mM for each forward and reverse), 2.4 µL of MgCl_2_, 2 µL of extracted DNA, and water to make up the volume of 20 µL. DNA amplification was carried out with the following run conditions: Pre-incubation for 5 min at 95 °C, followed by 45 cycles of amplification with denaturation at 95 °C for 30 s, annealing and extension for 1 min at 60 °C, and then a single cycle of cooling for 30 s at 40 °C^11^. Absolute quantification was carried out using the Light Cycler software 4.05. Positive control strains were used in the rPCR run (Table 4) These were obtained from the National Institute of Communicable Diseases (NICD), Johannesburg, South Africa.

**Table 3:** Primer sequences for detection of *bla*_CTX-M_, *bla*_SHV_, *bla*_TEM_, *bla*_IMP,_ *bla*_VIM_ genes and gyrB.

Target gene	Primers	Primers sequences (5′–3′)	Tm in 0 °C	References
*bla*_CTX-M_	CTX-M forward primer	ATGAGYACCAGTAARGTKATGGC	58.7	^45^
	CTX-M reverse primer	ATCACKCGGRTCGCCIGGRAT	59.3	^45^
	CTX-M Probe	FAM-CCCGACAGCTGGGAGACGAAACGT-BBQ	70.2	^45^
*bla*_SHV_	SHV forward primer	TCCCATGATGAGCACCTTTAAA	56.8	^46^
	SHV reverse primer	TCCTGCTGGCGATAGTGGAT	58.6	^46^
	SHV Probe	FAM-TGCCGGTGACGAACAGCTGGAG-BBQ	68.3	^46^
*bla*_TEM_	TEM forward primer	GCATCTTACGGATGGCATGA	56.6	^46^
	TEM reverse primer	GTCCTCCGATCGTTGTCAGAA	57.7	^46^
	TEM Probe	FAM-CAGTGCTGCCATAACCATGAGTGA-BHQ1	62.2	^46^
*bla*_IMP_	IMP forward primer	GGGCGGAATAGAGTGGCTTA	57.6	^47^
	IMP reverse primer	GGCTTGAACCTTACCGTCTTTTT	59.3	^47^
	IMP Probe	FAM-CGATCTATCCCCACGTATGCATCTGAATTAACA-BHQ1	67.4	^47^
*bla*_VIM_	VIM forward primer	TGCGCTTCGGTCCAGTAGA	59.0	^47^
	VIM reverse primer	TGACGGGACGTATACAACCAGAT	58.5	^47^
	VIM Probe	FAM-CTTCTATCCTGGTGCTGCGCATTCG-BHQ1	67.6	^47^
*gyr*B	*gyr*B forward primer	CCT GAC CAT CCG TCG CCA CAA		^48^
	*gyr*B reverse primer	CGC AGC AGG ATG CCG ACG CC		^48^
	*gyr*B probe	6-FAM-CCG TGG TGG TAG ACC TGT TCC CAG ACC-BHQ 6-FAM-CCG TGG TGG TAG ACC TGT TCC CAG ACC-BBQ		This study

**Table 4:** Control strains used for rPCR amplification.

Organism	ATCC/NCTC number	Inherent resistant gene
*Pseudomonas aeruginosa*	ATCC 27853	*gyrB*
*Escherichia coli*	NCTC 13461	*bla*_CTX-M_
*Klebsiella pneumoniae*	ATCC 700603	*bla*_SHV_
*Escherichia coli*	NCTC 13351	*bla*_TEM_
*Escherichia coli*	NCTC 13476	*bla*_IMP_
*P. aeruginosa*	NCTC 13437	*bla*_VIM_

### Statistical analysis

The data was coded and entered into a database on an Excel spreadsheet and analyzed using Statistical Package for the Social Sciences (SPSS) version 23.0. The descriptive analysis was performed to calculate the frequency and categorical variables were expressed as proportions (%). All statistical analysis was done with statistical significance set at ≤ 0.05.

### Ethics approval and consent to participate

Ethical approval for the study was granted by the Health Research Ethics and Biosafety Committee of the Faculty of Health Sciences, Walter Sisulu University (WSU) bearing the reference number 024/2016 while permission to conduct the study was obtained from the National Health Laboratory Services (NHLS). Informed consent was obtained from all study participants aged 18 years and above. However, for participants aged below 18 years, the consent was sought from a parent and/or legal guardian.

## Data Availability

All data generated or analysed during this study are included in this published article.
